# Rasch Measurement Model Supports the Unidimensionality and Internal Structure of the Arabic Oswestry Disability Index

**DOI:** 10.3390/jcm14041259

**Published:** 2025-02-14

**Authors:** Ali H. Alnahdi, Abdulrahman M. Alsubiheen, Mishal M. Aldaihan

**Affiliations:** 1 Department of Rehabilitation Sciences, College of Applied Medical Sciences, King Saud University, P.O. Box 10219, Riyadh 11433, Saudi Arabia; aalsubiheen@ksu.edu.sa (A.M.A.); mishaldaihan@ksu.edu.sa (M.M.A.); 2 King Salman Center for Disability Research, Riyadh 11614, Saudi Arabia

**Keywords:** psychometric, measurement property, structural validity, item response, patient-reported outcome measures

## Abstract

**Background/Objectives:** The objective of this study was to assess the unidimensionality and internal structure of the Arabic version of the Oswestry Disability Index (ODI) in patients with lower back pain (LBP) using the Rasch measurement model. **Methods:** Patients with LBP (N = 113) completed the Arabic ODI during their first visit to physical therapy departments. The Arabic ODI was examined by assessing its fit to the requirements of the Rasch measurement model. Chi-square statistics for item–trait interaction alongside mean item and person fit residuals were used for overall model fit assessment. Additionally, the analysis included assessments for the fit of individual items, the sequence of thresholds, local dependency, unidimensionality using the *t*-test method, and differential item functioning (DIF) by sex, age, chronicity, and the presence of radiating pain. **Results:** The overall fit of the Arabic ODI to the Rasch measurement model was supported by non-significant Chi-square statistics (χ^2^ = 25.32, *p* = 0.19) and acceptable mean item and person fit residuals. All items showed acceptable fit (standardized fit residual −1.89 to 1.62) with no violation of local item independence. The t-test method supported the scale’s unidimensionality. The ODI showed good internal consistency with a person separation index of 0.85, with good overall targeting of item thresholds to the participants’ lower back function. Items 2, 7, and 10 showed disordered thresholds, and potential bias by sex was detected in item 9 (social life). **Conclusions:** The Arabic ODI is a unidimensional measure valid for assessing disability due to low back pain; however, indications of the inappropriate functioning of some response options along with potential bias by sex need to be revisited.

## 1. Introduction

Lower back pain (LBP) is a significant public health issue and a leading cause of disability worldwide, affecting approximately 8.2% of the global population [1,2]. As a result, various disability assessment tools have been developed, with the Oswestry Disability Index (ODI) being one of the most widely used instruments for measuring disability related to LBP [3,4,5]. The ODI assesses a patient’s perceived limitations in daily activities, such as personal care, lifting, walking, and social life, and has been extensively validated across different languages and cultures [6,7,8].

The ODI’s Arabic version has been introduced with evidence supporting its reliability and construct validity but with concerns regarding its unidimensionality [9,10]. A recently published study supported the unidimensionality of the Arabic ODI using confirmatory factor analysis [11]. However, the internal structure of the Arabic ODI has not yet been evaluated using more rigorous modern measurement models such as the Rash measurement model. The term internal structure is used to refer to aspects of internal characteristic of the scale items in terms of the fit of individual items to the expectation of the measurement model, the behavior and appropriateness of the items’ response categories, local item independence, differential item functioning, and internal consistency. Additionally, no prior study has assessed the functioning of the scale response categories or the presence of item bias across important patient characteristics, nor delineated the hierarchy of item difficulty within the Arabic ODI and how Arabic-speaking patients with LBP perceive the difficulty of the different items.

Evaluating the internal structure of an assessment scale is crucial for ensuring its validity and fairness in measuring the intended construct. A well-defined internal structure guarantees that individual items align with the expectations of the measurement model, confirming that they collectively measure a single underlying trait rather than unrelated dimensions [12,13]. Proper response category functioning ensures that rating scales are interpreted consistently by respondents, minimizing ambiguity and enhancing measurement precision. Local item independence is vital, as dependencies between items can distort scores and lead to redundant information, compromising a scale’s ability to provide distinct, meaningful results [14]. Additionally, assessing differential item functioning helps identify potential biases in how different subgroups (e.g., by sex, age) respond to items, ensuring the scale is equitable and applicable across diverse populations. Finally, adequate internal consistency indicates that all items contribute to a coherent and stable measurement of the construct, reinforcing the scale’s reliability. Together, these elements of internal structure are essential for developing and validating a robust measurement tool that provides accurate, interpretable, and generalizable results across varied clinical and research settings [14,15].

The Rasch measurement model is a probabilistic measurement model that ensures that a scale measures a single underlying construct in a valid and reliable way [14,15]. It is based on the principle that an individual’s response to an item is governed solely by the item’s difficulty and the responder’s ability, making it independent of sample characteristics. The Rasch measurement model is particularly suited for evaluating the structural validity of instruments like the ODI [16]. One of the model’s key strengths is its ability to assess whether a scale’s items fit a single underlying construct, thereby confirming unidimensionality. Additionally, the Rasch model evaluates the internal structure of the scale, including the scoring structure of individual items, ensuring that the response categories are properly ordered. It also assesses local item independence, meaning that the response to one item should not directly influence the response to another, a key assumption in many measurement theories [17,18]. Additionally, the Rasch measurement model is well suited to assess for any differential item function or item bias and allow for the transformation of ordinal scores into interval-level scores, providing a more precise and meaningful representation of the underlying construct [19,20].

Given the lack of examination of the Arabic ODI using rigorous modern measurement models, this study aimed to use the Rasch measurement model to assess the unidimensionality and internal structure of the Arabic ODI, including evaluations of unidimensionality, individual item fit, response category functioning, local item independence, and potential item bias. This study also aimed to determine item difficulty hierarchies and whether items’ difficulty levels are well targeted to the disability level of patients with LBP.

## 2. Materials and Methods

### 2.1. Study Design

This cross-sectional study used the Rasch measurement model to evaluate the unidimensionality and internal structure of the Arabic version of the ODI. The study protocol was reviewed and approved by the institutional review boards at King Saud University (E-20-5529) and the Security Forces Hospital (22-601-37), and informed consent was obtained from all the participants.

### 2.2. Setting and Participants

This study was conducted across two physical therapy departments in the Security Forces Hospital and the Alrass General Hospital, which are located in the central region of Saudi Arabia, involving patients with LBP on their first clinic visit. Eligible participants were aged 18 years and older, had been referred to physical therapy for LBP, and were fluent in the Arabic language. Individuals were evaluated by specialist physicians in primary care, orthopedic, and spinal clinics and subsequently referred to physical therapy. Participants with systemic disease or neurological and cardiopulmonary disorders that were self-perceived to cause functional limitations were excluded from this study to exclude factors other than lower back pain causing functional limitation and disability. Participants who had previously undergone back surgery were also excluded from this study.

### 2.3. Procedure

Participants were asked to complete the Arabic ODI during their first physical therapy clinic visit. Relevant medical history, demographic data, including age and sex, chronicity of LBP (acute < 1 month; subacute 1–3 months; chronic > 3 months), and presence of radiating pain were also recorded at the same visit.

### 2.4. Outcome Measures

#### Oswestry Disability Index (ODI)

The Arabic version of the ODI was used as the primary outcome measure [9]. The ODI consists of 10 items, each scored on a 6-point Likert scale ranging from 0 to 5, where 0 represents no disability and 5 represents maximum disability. The recommended total scoring method for the ODI involves adding all items scores and then convert this sum into a percentage (0–100), with higher scores indicating greater disability [6,7]. Previous studies have examined the reliability and construct validity of the Arabic version of the ODI, supporting these measurement properties for the Arabic ODI [9,10].

### 2.5. Statistical Analysis

Rasch analysis in the current study was conducted using the partial credit model [21] and the specialized software RUMM2030 [22]. Rasch analysis addressed the objectives of this study by assessing the unidimensionality and internal structure (fit of individual items to the expectation of the measurement model, the appropriateness of items’ response categories, local item independence, differential item functioning, and internal consistency) of the Arabic ODI. The details of how these aspects were assed are detailed below. The item–trait interaction was used to assess the overall fit of the observed data to the Rasch measurement model, with a non-significant Chi-square statistic (*p* > 0.05) was used to indicate good fit [16,22]. The overall fit was evaluated by examining the item and person fit residuals. Good overall fit to the Rasch measurement model was indicated when the mean item and person fit residuals were near zero, and the standard deviation was approximately one. Good overall fit of the Arabic ODI would support the notion that the Arabic ODI satisfies the requirement of the Rasch measurement model and that responses to the ODI items are governed solely by the item’s difficulty and the responder’s ability. In addition to the assessment of overall model fit, the fit of each item and person was evaluated by assessing the standardized fit residuals. Standardized fit residuals exceeding ± 2.5, along with significant Chi-square statistics (adjusted using Bonferroni correction), were considered indicators of poor fit of an individual item to the Rasch measurement model [16,22]. The scoring structure of Arabic ODI items was assessed to ensure that each response category functioned as intended. Threshold ordering was evaluated by assessing individual item category characteristic curves, with disordered thresholds indicating that participants could not distinguish between adjacent response categories [16,18,23]. To check for local item independence, the residual correlations between items were analyzed. Items with residual correlations exceeding 0.2 were flagged for potential local dependency [12,24]. To assess the unidimensionality of the Arabic ODI, the t-test method was applied [16,25,26]. The process began with a principal component analysis (PCA) of the residuals. The loadings of the first component were then used to classify the items into two groups: those with positive loadings and those with negative loadings. Using these groups, the participants’ LBP-related disability was estimated twice, once for each set of items. The two estimates were then compared through a t-test. The Arabic ODI was deemed unidimensional if the proportion of significant differences between the estimates did not exceed 5% [16,25]. The performance of the Arabic ODI’s items was analyzed to determine whether they functioned consistently across different patient subgroups [13,16]. Differential item functioning (DIF) was assessed based on sex (male vs. female), age (younger than 28 vs. 28 or older, based on the median), chronicity (acute and subacute vs. chronic), and the presence of radiating pain (yes vs. no). To identify both uniform and non-uniform DIF, residuals were analyzed using two-way ANOVAs, factoring in class intervals and subject characteristics. The person separation index was used to examine the internal consistency of the ODI. Targeting of the ODI items to the ability level of the participants was examined by assessing the person–item threshold plot where, in a well-targeted test, the range of item thresholds should overlap with the range of a person’s abilities.

### 2.6. Sample Size Estimation

The necessary sample size was established in accordance with the COSMIN guidelines [27]. A cohort of 100 individuals was deemed sufficient by the COSMIN criteria for the assessment of structural validity (unidimensionality) utilizing Rasch analysis; therefore, a minimum sample size of 100 was employed in the present investigation.

## 3. Results

A total of 113 patients with LBP were recruited in the current study. The characteristics of the participants are summarized in Table 1. In the present investigation, the analysis was performed utilizing the partial credit model, as the likelihood ratio test [22] had yielded a significant result (*p* < 0.001), suggesting that the partial credit model [21] would be more suitable than the rating scale model [28].

The first two runs of the analysis identified misfitting individuals that were then removed from the analysis (Table 2). Assessments of the Arabic ODI’s overall fit after the removal of misfitting individuals indicated good fit to the Rasch measurement model (Table 2). The Chi-square statistics for item–trait interactions was non-significant, indicating a good overall fit to the Rasch model. The mean item and person fit residuals were close to zero, with acceptable standard deviations near 1 supporting the overall fit the ODI to the Rasch model (Table 2).

The individual ODI items showed good fit to the requirements of the Rasch measurement model. This was supported by the acceptable individual item standardized fit residuals, ranging from −1.89 to 1.62, with non-significant Chi-square statistics (Table 3). The t-test methodology revealed that a relatively low proportion of the subjects exhibited a statistically significant distinction between the two estimates of the disability level (derived from the two test sets), thereby corroborating the unidimensional nature of the scale (Table 2). This conclusion was based on the lower limit of the binomial 95% confidence interval for the percentage of significant t-tests, which was less than 5%. (Table 2). The assessment of residual correlations between items revealed no two pairs with residual correlation above the pre-determined threshold (0.2), suggesting no breach of the local dependency between these items. The highest residual correlation was observed between item 4 (walking) and item 6 (standing) (r = 0.169).

**Figure 1:**
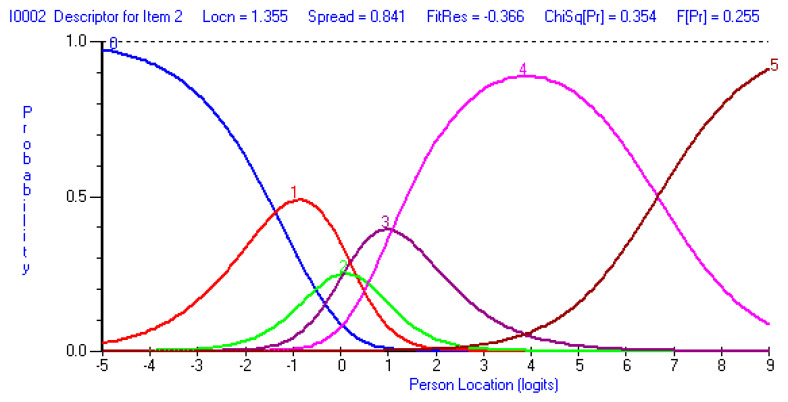

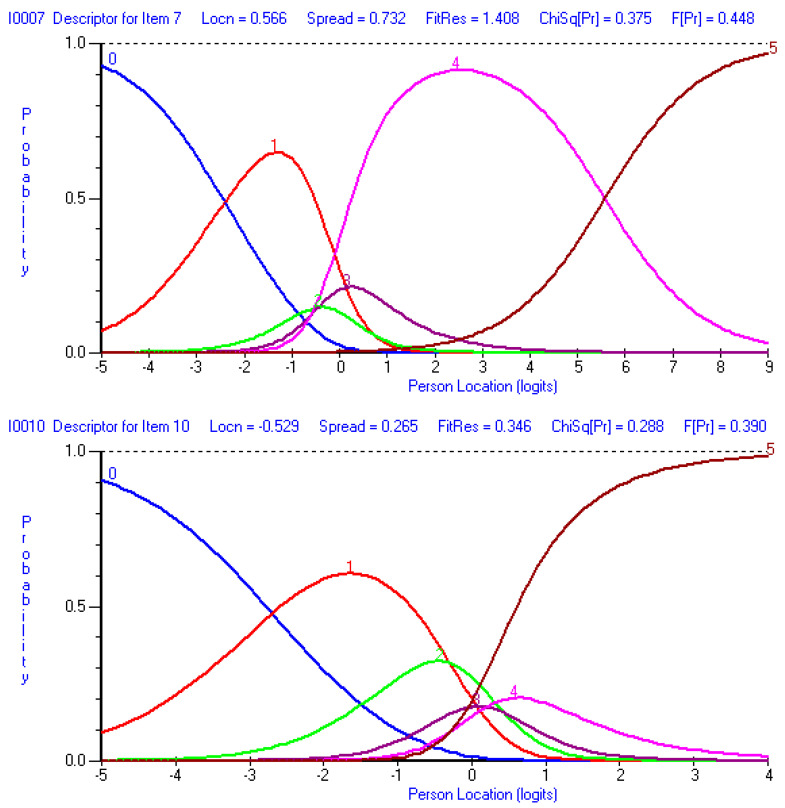
Category characteristic curves for the ODI items exhibiting disordered thresholds: item 2 (personal care, **top figure**); item 7 (sleeping, **middle figure**); and item 10 (traveling, **bottom figure**). Category characteristic curves were assessed for all items to examine whether the response categories displayed proper function and sequence. Lines 0 to 5 represents the six response categories for each ODI item with where 0 represents no disability and 5 represents maximum disability.

**Figure 2:**
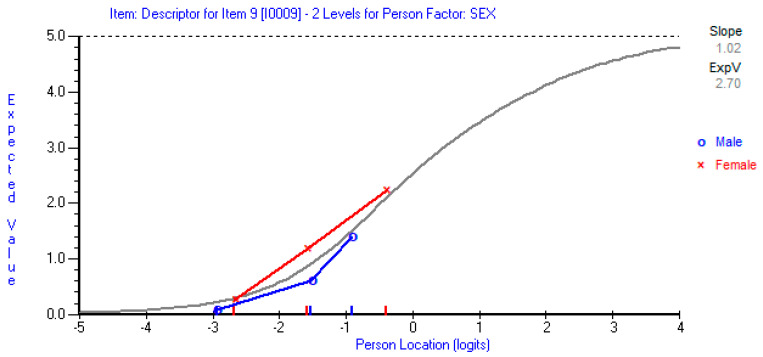
Item characteristic curves for item 9 (social life), showing the differential function of this item in male versus female respondents despite similar levels of lower back function.

**Figure 3:**
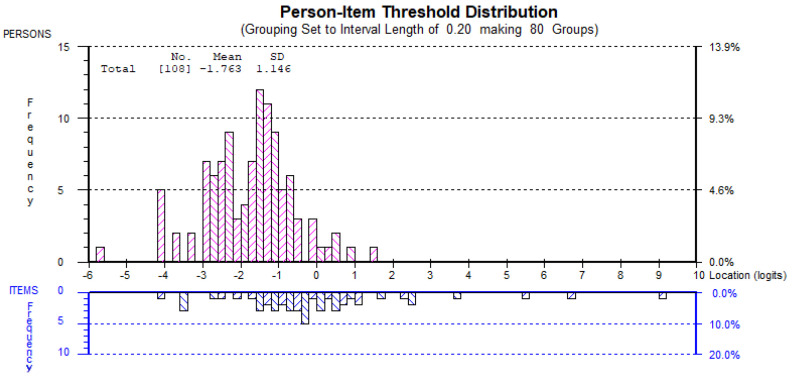
Person–item threshold plot showing the good spread of item thresholds and overlap with the range of person abilities, except in those with a very high level of lower back function (lower than −5 logit). This figure assessed the targeting of the ODI’s items to the ability level of the participants.

Despite the good fit of the Arabic ODI to the requirements of the Rasch measurement model, some deviations from the model were observed. Items 2 (personal care), 7 (lifting), and 10 (social life) exhibited some threshold disordering, suggesting the inappropriate use of the response categories for these items (Figure 1). A uniform DIF was detected for item 9 (social life) based on sex, with females perceiving greater difficulty in social life than males (*p* < 0.001) (Figure 2). The ODI showed good internal consistency with a person separation index of 0.85. The range of ODI item thresholds exhibited good spread and overlap with the range of person abilities, except in those with a very high level of function (lower than −5 logit), generally suggesting acceptable targeting to the intended population (Figure 3). ODI items’ difficulty hierarchy is displayed in Table 3 with pain intensity and then lifting determined to be the most difficult items and walking, followed by personal care, being the easiest.

## 4. Discussion

Using the Rasch measurement model, the current study confirmed the unidimensionality and appropriate internal structure of the Arabic ODI, indicating that it assesses a single underlying construct related to disability due to LBP. This finding suggested that Arabic ODI is a valid measure for assessing disability and functional limitations in Arabic-speaking patients with LBP, which aligns with the intended purpose of the instrument.

The Arabic ODI demonstrated an excellent overall fit with the Rasch measurement model. This fit suggests that the Arabic ODI could provide accurate interval-level measurement, allowing clinicians and researchers to capture lower back disability levels effectively across a spectrum of LBP severity. In Rasch models, item difficulties should ideally remain stable (invariant) across the entire range of person abilities, meaning that item performance should not vary for different ability groups [29]. The non-significant item–trait interaction reported in our study suggested that the Arabic ODI scale was functioning consistently across all levels of lower back function, with only a single underlying construct (lower back function) governing the functioning of all items [16]. A significant item–trait interaction, on the other hand, would have indicated potential misfit between the data and the Rasch model, suggesting that the given scale does not measure the construct consistently across different levels of disability [16]. This could result in measurement bias, where certain items function differently depending on the severity of the condition, ultimately affecting the reliability and validity of the instrument. The absence of such interaction in our findings reinforces the Arabic ODI’s suitability for use in diverse clinical populations, ensuring that scores can be meaningfully compared across individuals with varying levels of lower back disability.

Individual items within the Arabic ODI exhibited acceptable standardized fit residuals suggesting that the functioning and scores of the ODI’s individual items were only governed by one construct—lower back function—and that each item contributed effectively to assessing the overarching construct. The good fit exhibited by all the items within the Arabic ODI suggests that these item behaved in line with the expectation of the Rash measurement model, where the response to the item was governed by the individual’s level of lower back function and the difficulty of the item, meaning that participants with lower back function higher than the difficulty of the item would end up reporting a higher probability of being able to perform the activity without difficulty. These findings are consistent with previous studies that explored the ODI’s item-level fit and reported adequate fit to the Rasch measurement model [30,31,32,33]. Additional studies indicated that ODI items showed good fit to the Rasch model, except for the pain intensity item, which was determined to be misfitting [34,35]. The ODI’s versions used in these studies contain a pain intensity item related to the use of painkillers and pain relief rather than pain intensity. This difference in item content suggests that variations in how pain is framed within the questionnaire may influence its measurement properties and overall model fit. Specifically, assessing pain intensity through medication use introduces additional factors, such as differences in prescription practices, medication accessibility, and individual responses to pain relief methods, which may not directly reflect the severity of the pain itself.

Threshold disordering in three items (items 2, 7, and 10) was observed in our study, suggesting potential issues with the response scale functioning for these items. Disordered thresholds often reflect difficulty in distinguishing between adjacent response categories, potentially compromising measurement precision for those items. This issue may arise if respondents struggle to differentiate between response options due to ambiguous wording, overlapping severity levels, or inconsistencies in how they interpret the scale. Disordered thresholds in ODI items have been reported in previous studies [30,32,33,34,35], but the number of items exhibiting disordered thresholds along with the specific dysfunctional response categories vary among these. To address the issue of disordered thresholds, a couple of previous studies recommended and implemented a general change to the ODI item-scoring scheme, from a six-point score to either a five-point score [32] or a four-point score [34,35]. A replication of the findings of the current study is needed to ensure consistency of the observed disordered thresholds before recommending and implementing changes to the scoring scheme of an established outcome measure such as the ODI. Ensuring that all items exhibit ordered thresholds will enhance the precision of the Arabic ODI, making it a more effective tool for assessing disability severity and tracking functional changes over time.

The current study detected DIF in item 9 (social life) based on sex, with female participants perceiving it as more challenging than their male counterparts at similar disability levels. This DIF could stem from cultural or societal factors influencing social engagement perceptions in Arabic-speaking populations, particularly among women. It should be made clear that the DIF analysis conducted in the current study should be considered a preliminary analysis with preliminary findings given that the number of participants within each subgroup was below the recommended sample size [27]. Thus, a larger sample is needed to draw a firmer conclusion regarding DIF in the Arabic ODI. Most previous studies did not examine for DIF in the ODI items by age, sex, or any other relevant patient characteristics [31,32,34,35]. Two previous studies reported no DIF by sex in the ODI items but reported uniform DIF by age in item 4 (walking) [33] and item 7 (sleeping) [30]. Based on these studies, it seems that there is no consistent DIF pattern among the published studies. Given these mixed findings, future research should prioritize more extensive DIF analyses with adequately powered samples to determine whether the DIF in item 9 is a stable characteristic of the Arabic ODI or a sample-specific occurrence. If confirmed, the presence of DIF in the social life item may indicate that modifications are necessary to enhance measurement fairness across sex groups, such as revising the item’s wording or exploring alternative response formats. Additionally, DIF should be assessed across other demographic and clinical variables, such as age, education level, and pain duration, to ensure that the Arabic ODI functions equitably across diverse patient populations.

Pain intensity and lifting emerged as the most difficult items. Following these were items associated with prolonged standing and traveling, which were moderately difficult. Items related to sexual function, social life, and sleeping represented intermediate levels of difficulty. Meanwhile, items focused on personal care and walking were identified as the easiest in this hierarchy, suggesting that patients with LBP perceived these activities as more manageable. Similar to the current study, lifting was consistently reported in the literature to be among the most difficult items in the ODI and personal care was consistently reported to be among the easiest items [30,33,34,35]. Page et al. reported walking to be among the easiest items in the ODI, similarly to our finding [35]. We reported pain intensity to be the most difficult item, while two previous studies ranked pain intensity among the items with medium difficulty [30,33]. This indicates that the Arabic-speaking patients with LBP in our study perceived pain intensity to be a more difficult item in comparison to patients from different cultures. Further research is needed to explore whether these difficulty rankings remain consistent across different Arabic-speaking regions and whether factors such as the chronicity of LBP, occupational demands, and gender differences influence item difficulty perception. Understanding these variations can help refine the interpretation of ODI scores and improve the scale’s applicability in diverse clinical settings. Additionally, examining how item difficulty rankings change over time with treatment interventions could provide valuable insights into the responsiveness of the Arabic ODI and its utility in tracking patient progress.

A major strength of this study was its rigorous Rasch analysis, which validated the Arabic ODI’s structure and item fit. This validation supports clinical and research applications in Arabic-speaking populations with LBP, providing a valid tool for assessing disability. However, this study’s limitations include its specific focus on patients within Saudi Arabia, which may limit generalizability to Arabic speakers from other regions. The findings of the DIF analysis in this study should be interpreted with caution giving that dividing the full sample into subgroups for DIF analysis (e.g., male and female) resulted in each subgroup having fewer participants than recommended for scale invariance assessment [27]. Furthermore, the disordered thresholds suggest potential response clarity issues, which could be addressed in future research. Recommendations for further studies include exploring refined response categories for items with disordered thresholds and evaluating the DIF findings to enhance the scale’s universality and interpretative accuracy.

## 5. Conclusions

The Rasch measurement model confirmed the unidimensionality and appropriate internal structure of the Arabic ODI, indicating that it assesses a single underlying construct reflecting low back pain-related disability. However, the Arabic ODI demonstrated inappropriate functioning of response categories in three items and indication of bias by sex in one item.

## Figures and Tables

**Table 1:** Characteristics of the participants (N = 113).

Variable	Mean ± SD or N (%)
Age (year)	30.45 ±12.07
Age group	
≤28 years	57 (50.44)
>28 years	56 (49.56)
Sex	
Male	54 (47.8)
Female	59 (52.2)
Height (m)	1.67 ± 0.10
Mass (Kg)	71.54 ±12.14
Body mass index (Kg/m^2^)	25.96 ± 5.61
LBP duration	
Acute (<1 month)	17 (15.04)
Subacute (1–3 months)	19 (16.81)
Chronic (>3 months)	77 (68.14)
Radiating pain	
Yes	78 (69.03)
No	35 (30.97)
Oswestry Disability Index (0–100)	25.27 ± 15.38

LBP = Lower back pain.

**Table 2:** ODI Rasch analysis results at each run.

Run	Analysis	N	Item Fit Residual		Person Fit Residual		Item–Trait Interaction		PSI	Unidimensionality T-Tests	
			Mean	SD	Mean	SD	χ^2^ (df)	*p*		% of Significant Tests	Lower Limit of 95% CI *
1	Initial analysis	113	0.249	1.329	−0.228	1.098	26.02 (20)	0.165	0.86	8.85%	4.80%
2	Delete 4 misfit persons	109	0.205	1.26	−0.193	0.973	25.94 (20)	0.168	0.85	7.34%	3.20%
3	Delete 1 misfit person	108	0.191	1.242	−0.187	0.945	25.32 (20)	0.189	0.85	6.48%	2.40%

ODI = Oswestry Disability Index; SD = standard deviation; χ^2^ = Chi-square; df = degrees of freedom; and PSI = person separation index. * Represents the lower limit of the binomial 95% confidence interval for the percentage of significant *t*-tests.

**Table 3:** Items’ hierarchy and calibration of the ODI after removing misfit individuals (items arranged from the most difficult to the easiest).

Item	Location	SE	Fit Residual	χ^2^	*p*	Ordered Thresholds
1 (Pain intensity)	−1.15	0.10	1.46	3.01	0.22	✓
3 (Lifting)	−1.10	0.11	0.58	3.73	0.15	✓
6 (Standing)	−0.83	0.11	0.56	0.12	0.94	✓
10 (Traveling)	−0.53	0.12	0.35	2.49	0.29	×
5 (Sitting)	−0.17	0.12	1.62	1.41	0.49	✓
8 (Sex life)	0.12	0.12	−1.75	6.62	0.04	✓
9 (Social life)	0.16	0.12	−1.89	3.53	0.17	✓
7 (Sleeping)	0.57	0.13	1.41	1.96	0.37	×
2 (Personal care)	1.36	0.14	−0.37	2.08	0.35	×
4 (Walking)	1.58	0.12	−0.04	0.37	0.83	✓

ODI = Oswestry Disability Index; SE = standard error; and χ^2^ = Chi-square. ✓ = indicate all thresholds were ordered; × = indicate the existence of disordered thresholds.

## Data Availability

The data presented in this study are available from the corresponding author upon reasonable request.
